# Low serum phosphate levels are related to increased cardiovascular risk in HIV-1 infected patients

**DOI:** 10.1186/1758-2652-13-S4-P66

**Published:** 2010-11-08

**Authors:** P Grima, M Guido, R Chiavaroli, P Tundo, A Zizza

**Affiliations:** 1 S. Caterina Novella Hospital, Division of Infectious Diseases, HIV Centre, Galatina, Italy; 2 University of Salento, Di.S.Te.B.A., Faculty of Science, lecce, Italy;; 3 National Research Council, Institute of clinical Physiology, lecce, Italy

## Purpose of the study

Hypophosphatemia may contribute directly to the development of obesity, hypertension and dyslipidemia. Hyperglycemia, insulin resistance, hyperlipidemia and hypertension, which are components of metabolic syndrome, are also recognized as strong risk factors for cardiovascular disease [1]. This study was performed to determine whether serum phosphate levels are associated with increased risk for cardiovascular events.

## Methods

We enrolled 125 consecutive HIV-1-infected patients in a cross-sectional study. All patients were receiving highly active antiretroviral therapy (HAART) for more than six months. Fasting phosphate, lipids (cholesterol, HDL, triglycerides), Homeostasis Model Assessment (HOMA), blood pressure were evaluated. Framingham 10 years risk of general cardiovascular disease was used to assess three cardiovascular risk (CVR) categories (low CVR < 10%, medium CVR between 10 and 20%, high CVR > 20%).

## Summary of results

**Figure 1:**
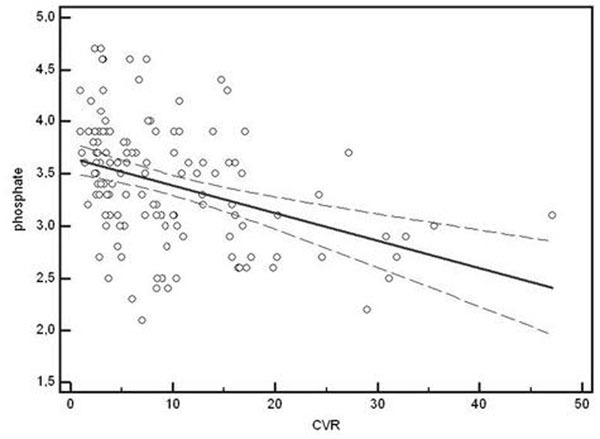


We observed a statistically significant decrease in serum phosphate levels in the three different CVR groups (low risk: 3.5 mg/dl; medium risk: 3.3 mg/dl; high risk: 2.9 mg/dl; p=0.001). There was a strong negative correlation between Framingham score and phosphate levels (r:-0.37, p<0.0001). Figure 1

Multiple regression analysis, including age, months of HAART, CD4 cells count, cholesterol, HDL, HOMA, systolic pressure, months of Tenofovir use, showed that only HOMA (r:-0.30, p<0.01) and age (r:-0.3, p<0.01) were the most important determinants of serum phosphate values.

## Conclusions

We found that lower phosphate level is correlated with cardiovascular risk and insulin resistance. Therefore, when serum phosphate levels are too low the patients is at risk for cardiovascular events and/or metabolic syndrome.
